# Discussion of Dr. Claude Smith’s Paper on “Diptheria, Its Diagnosis, Etc.”

**Published:** 1912-12

**Authors:** 


### DISCUSSION OF DR. CLAUDE SMITH'S PAPER ON "DIPHTHERIA, ITS DIAGNOSIS, ETC."

We had an epidemic of diphtheria in Colon, Panama, a year ago. As the cases became more and more numerous, the Sanitary Department decided to make every effort to discover and isolate all *diphtheria carriers* also. When this decision had been reached, however, it was discovered that there was quite a shortage of culture tubes. Dr. Zeiler stated that he had read in a recent medical journal that some one considered more or less an authority, had stated that in most *dipththeria carriers* examination would reveal one or all of following signs: areas of conjested, reddened mucosa or slight hemorrhage aveas in nose or throat. He suggested that the eye and ear clinic staff with the aid of his staff carry out systematic examination of all children of Colon, and take cultures only of those whose noses or throats showed these lesions. The Sanitary Department agreed and the alcolde (mayor) of Colon issued an order which compelled all children to be brought to us for this examination. We devoted every afternoon for about two weeks to this work. We examined a total of exactly 3,656 children. Cultures and smears were taken from only 104 children. All cultures and smears were negative. Two children of those from whom cultures had not been taken, subsequently developed diphtheria.

We concluded naturally, that such a method for discovering diphtheria carriers is absolutely valueless.

